# Eating disorders among adolescents and young adults in Kingston, Jamaica

**DOI:** 10.3389/fpsyg.2026.1704895

**Published:** 2026-02-23

**Authors:** Abigail Harrison, Caryl James, Kimberley Ferguson-Henry, Kern Rocke, Gillian Lowe

**Affiliations:** 1 Department of Child and Adolescent Health, The University of the West Indies, Kingston, Jamaica; 2 Department of Sociology, Psychology and Social Work, Faculty of Social Sciences, The University of the West Indies, Mona Campus, Kingston, Jamaica; 3 The University of the West Indies, Kingston, Jamaica; 4 George Alleyne Chronic Disease Research Centre, The University of the West Indies at Cave Hill, Bridgetown, Barbados; 5 Department of Community Health and Psychiatry, The University of the West Indies, Mona Campus, Kingston, Jamaica

**Keywords:** adolescents, young adults, eating disorders, Jamaica, management, outcomes

## Abstract

**Introduction:**

Eating disorders are complex disorders characterized by a persistent disturbance of eating that impairs health and psychosocial functioning, with accompanying long-lasting effects on the individual’s overall health and well-being. Presumed stereotypes may affect reporting and access to care, especially in the Caribbean, where ethnic groups are mostly non-White. This study reviewed the clinical presentation, management, and outcomes of eating disorders in adolescents and young adults in Kingston, Jamaica. It explored the factors that influence outcomes – hospital admission and recovery.

**Methods:**

This retrospective case review study included male and female adolescents and young adult participants (10–29 years old) being treated for an eating disorder in Kingston, Jamaica. Data were extracted from the medical records of patients meeting the inclusion criteria for the period January 2010 to December 2020. Sociodemographic data, medical and psychological symptoms and signs, management and outcomes were extracted. Descriptive analyses were performed - proportions and frequencies for categorical variables; means and medians for continuous variables. Inferential analyses including the independent student’s t-test and analysis of variance were used to compare means and the Pearson chi-squared test used to determine the association between categorical variables. Logistic regression analyses examined predictors of outcome. Analyses were performed using SPSS version 23. Statistical significance was determined at the 5% level.

**Results:**

The study included 68 participants with a female preponderance (*n* = 60, 88.2%). Other specified food and eating disorders (OSFED) (*n* = 27, 39.7%) was the most common subtype seen in the sample population, and included atypical anorexia nervosa (AAN) (*n* = 18, 26.5%). Eleven (16.2%) participants were admitted to the hospital, the majority diagnosed with OSFED (*n* = 6, 54.5%). The most common reason for admission was failure of outpatient management (*n* = 7, 63.6%) but also included admissions for active suicidal ideation (*n* = 1) and late presentation of significant severity (*n* = 2). Multidisciplinary management by an ED-trained physician, ED-psychologist, and dietician was utilized in most participants.

**Discussion:**

Eating disorders are an emerging health concern worldwide, as well as in Jamaica. OSFED with a preponderance of atypical anorexia nervosa is the most prevalent, with overall female preponderance. These findings necessitate the implementation of measures in Jamaica in the realms of identification, prevention, and treatment.

## Introduction

Eating disorders (EDs) are complex biopsychosocial disorders characterized by a persistent disturbance of eating which impairs health and psychosocial functioning that usually have their onset during adolescence (Golden et al., 2015). Eating disorders may be considered psychiatric illnesses with potential for grave medical consequences with significant morbidity affecting multiple systems, and may even result in mortality. The prevalence of eating disorders - anorexia nervosa (AN), bulimia nervosa (BN), binge-eating disorder (BED), avoidant/restrictive food intake disorder (ARFID) and other specified feeding or eating disorder (OSFED), has increased over the decades globally (Galmiche et al., 2019; van Hoeken and Hoek, 2020). Despite this, EDs continue to be under-represented or misdiagnosed by professionals (van Hoeken and Hoek, 2020). A high index of suspicion is required, particularly among children and adolescents, where EDs may present differently (Campbell and Peebles, 2014). Eating disorders may present with a myriad of physical symptoms, ranging from vague abdominal symptoms such as constipation, a sense of bloating, to abdominal pain or vomiting; cardiovascular instability with near syncope or syncopal events, significant postural changes in pulse rate and or blood pressure, as well as arrythmias - sinus bradycardia being most common; headaches or difficulties concentrating.

Persons suffering from EDs may also have delayed presentation for treatment due to the persistent stigma regarding mental illness in general and by extension EDs, and even more so given the high prevalence of comorbid mental disorders, such as depression and anxiety with EDs (Rikani et al., 2013).

Inherent misunderstanding regarding the epidemiology of EDs may result in missed screening opportunities for disordered eating behaviors in certain sub-populations, such as people of color (Becker et al., 2003), and in countries where the resources to treat and where training are limited (Harrison et al., 2020). Sala and colleagues propose that American adolescents with eating disorders in the Black, Indigenous, People of Color (BIPOC) group may receive suboptimal care for varied reasons, inclusive of atypical presentations as well as a lower likelihood of screening for EDs by healthcare providers (HCPs). The latter may be due to the misconception of low prevalence among the BIPOC sub-population (Sala et al., 2013). Countries like Jamaica, with an ethnically diverse population and where the majority are of Afro-Caribbean descent, may have similar inherent misconceptions (Caribbean Community Secretariat, 2025).

There is a dearth of information in Jamaica related to the prevalence, incidence, and biopsychosocial effects of eating disorders in adolescence and beyond. A dated review of ED in Jamaica (1985–1998) concluded the prevalence of eating disorders was very low (White and Gardner, 2002). However, more recently, a rise in the prevalence of disordered eating behaviors and attitudes in Jamaican adolescents has been reported, with as many as 1 in 5 adolescents being considered ‘at-risk’ of developing an ED, comparable with some high-income countries (Harrison et al., 2020). More disconcerting are the limited local and regional healthcare providers specially trained in the management of EDs in adolescents, with no healthcare facilities designed specifically to treat these conditions and a paucity of local evidence-based guidance. This may leave patients and their families feeling unsupported (Johns et al., 2019) and practitioners ill-equipped at identifying and making the appropriate timely referrals.

This study sets out to achieve two major objectives: (1) to examine the clinical presentation, management, and outcomes of eating disorders among Jamaican adolescents and young adults (AYAs), 10–29 years old, and (2) to explore the factors that influence outcomes – hospital admission, and recovery.

This study sought to answer the following research questions:

1. What are the characteristics of the Jamaican patients who were diagnosed with an eating disorder?
2. What were the observed clinical features of the patients for each ED category?
3. What were the psychological symptoms that patients reported in addition to their ED?
4. What were the treatment outcomes of the ED patients seen?

## Methods

### Study design

A retrospective chart review study design was utilized to examine adolescent and young adult patients diagnosed with an eating disorder. Patients included in this chart review were those who were diagnosed with an eating disorder and received treatment at the University Hospital of the West Indies (UHWI) by the psychiatry and or adolescent medicine teams; as well as those patients seen privately by psychiatrists, adolescent medicine specialists, and psychologists, in the Kingston metropolitan region, Jamaica, from January 2010 to December 2020.

### Participants and sample size

**Figure 1:**
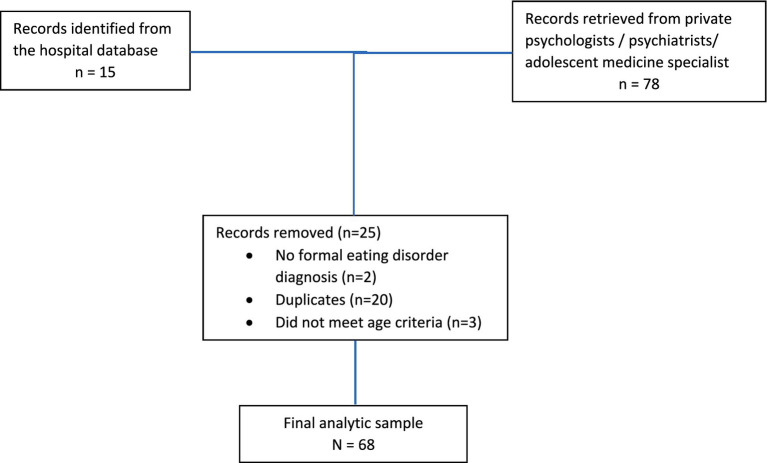
Flow diagram of participants.

The medical records department performed an electronic search for all the patients attending UHWI (inpatients and outpatients) with a primary or secondary diagnosis of anorexia nervosa, bulimia nervosa, binge eating disorder, eating disorder, or disordered eating, to ensure retrieval of all possible patients with an ED, including those that may have been mislabeled when recorded in the medical records system. Relevant soft or hard copy files were then retrieved from the records department for review by the researchers. Potential participants in the private practice setting in Kingston were identified by inviting the healthcare providers through their professional organisations via the usual communication route of email, to identify patients they had managed during the study period. The relevant charts were then made available for review by the researchers with the approval of the specific health care provider in their offices. All patients meeting the inclusion (formally diagnosed with an eating disorder; age 10 – 29 years) and exclusion criteria (no formal diagnosis of ED or outside age range) (see Figure 1).

### Measures

Data was extracted on questions such as lifetime diagnosis of psychiatric illness(s), sociodemographic characteristics, first consultation for ED and what prompted the consultation, psychiatric comorbidities, treatment histories [non-pharmacotherapy and pharmacotherapy] and treatment effects.

Participants were categorized by age – early adolescence (10–15y), late adolescence (16–19y), and young adulthood (20–29y); and nutritional status using body mass index (BMI) – underweight, normal, overweight, and obesity (Kuczmarski et al., 2000; Guo et al., 2002).

For participants less than 20 years old, the following definitions were used: Underweight: BMI < 5th centile for age; Normal: BMI 5th to < 85th centile; Overweight: BMI 85th – < 95th centile; Obesity: BMI ≥ 95th centile.

For patients aged ≥ 20 years, the following criteria were applied - Underweight: BMI < 18.5 kg/m^2^; Normal: BMI 18.5–24.9 kg/m^2^; Overweight: BMI 25–29.9 kg/m^2^; Obesity: BMI ≥ 30.

### Ethical considerations

The study protocol and data extraction form were reviewed and approved by the local Institutional Review Board (IRB). All the precautions were taken to maintain confidentiality and compliance with the Declaration of Helsinki. When applicable, patient/parent consent was obtained. All data collected were deidentified and saved electronically, with only researchers with direct responsibility for data entry and analysis having access to the data. Written consent was obtained from those participants who were minors (under 18 years) at the time of treatment, and assent for those who are still less than 18 years, along with parental consent.

### Statistical analysis

Descriptive analyses were performed using proportions and frequencies for categorical variables and means and medians to describe dispersion for continuous variables. Inferential analyses were performed using the Independent Student’s *t* test and analysis of variance as appropriate to compare means, and the Pearson Chi-squared test was used to determine the association between categorical variables. The relationship between hospital admission and defaulting from medical care follow-up was examined against predictors using logistic regression models. We first examined each predictor using a univariate model and then using a multivariable model adjusting for confounders such as sex, age, family history of comorbid mental health conditions, and personal mental health co-morbid conditions (anxiety/depression) and history of abuse. To account for missing data, we sought to conduct all our analyses using a complete case analysis approach. All analyses were performed using SPSS version 23, and statistical significance was determined at the 5% level.

## Results

A total of 68 patients diagnosed with an ED were reviewed, with a female preponderance (F: *n* = 60, 88.2%; M: *n* = 8, 11.8%). Thirteen (19.1%) participants were diagnosed with anorexia nervosa (AN); 18 (26.5%) with bulimia nervosa (BN); 7 (10.3%) with binge eating disorder (BED); 3 (4.4%) with avoidant restrictive food intake disorder (ARFID) and 27 (39.7%) with other specified food and eating disorder (OSFED) - atypical AN = 18 (26.47%); other OSFED = 9 (13.24%). The sample was comprised of early adolescents (*n* = 22, 32.4%), late adolescents (*n* = 32, 47.1%), and young adults (*n* = 14, 20.6%) which approached statistical significance regarding onset of DEBAs by ED diagnosis (*p* = 0.071) (Table 1). We found no statistically significant differences in the mean age of onset or age at formal diagnosis of an ED. We observed statistically significant associations between ED diagnosis and nutritional status (*p* = <0.001); family history of psychiatric illness (*p* = 0.012), and triggers for onset of ED (*p* = 0.028), with those patients diagnosed with OSFED being most likely to report family stressors as the trigger for their ED symptomatology. Eleven (16.2%) patients were admitted during the review period (AN = 4; BN = 1; OSFED = 6) secondary to failed outpatient management (*n* = 7); active suicidal ideation (*n* = 1); and severe presentation (biochemical derangement; cardiovascular instability) due to delayed diagnosis (*n* = 3). There were no deaths reported during the study period.

**Table 1:** Patient characteristics of sample population by ED diagnosis.

Characteristic	Total *N* (%)	AN *n* (%)	BN *n* (%)	BED *n* (%)	ARFID *n* (%)	OSFED *n* (%)	*p*-value
Sex							0.494
Male	8 (11.8)	1 (12.5)	2 (25.0)	2 (25.0)	1 (12.5)	2 (25.0)	
Female	60 (88.2)	12 (20.0)	15 (25.0)	6 (10.0)	2 (3.3)	25 (41.7)	
Age category at first presentation							0.071
Early adolescence (10–14 y)	22 (32.4)	3 (13.6)	3 (13.6)	2 (9.1)	0 (0.0)	14 (63.6)	
Late adolescence (15–19 y)	32 (47.1)	8 (25.0)	9 (28.1)	2 (6.3)	2 (6.3)	11 (34.4)	
Young adult (≥20 y)	14 (20.6)	2 (14.3)	5 (35.7)	4 (28.6)	1 (7.1)	2 (14.3)	
Mean age (years) at onset of DEBAs, mean (SD)	13.8 (2.6)	13.4 (2.7)	13.9 (1.8)	15.2 (4.6)	15.5 (0.7)	13.6 (2.6)	0.635
Mean age (years) at formal ED diagnosis, mean (SD)	15.7 (3.7)	16.0 (3.6)	17.2 (2.1)	18.8 (4.4)	15.5 (0.7)	14.5 (4.1)	0.117
Duration (years) onset → diagnosis, mean (SD)	1.9 (3.5)	2.7 (4.1)	3.3 (2.6)	3.5 (3.5)	0.0 (0.0)	0.9 (3.5)	0.212
Nutritional status on presentation							<0.001
Underweight	17 (25.0)	11 (64.7)	2 (11.8)	1 (5.9)	3 (17.6)	0 (0.0)	
Normal weight	37 (54.4)	2 (5.4)	10 (27.0)	2 (5.4)	0 (0.0)	23 (62.2)	
Overweight	6 (8.8)	0 (0.0)	4 (66.7)	0 (0.0)	0 (0.0)	2 (33.3)	
Obesity	8 (11.8)	0 (0.0)	1 (12.5)	5 (62.5)	0 (0.0)	2 (25.0)	
Sexual orientation							0.969
Heterosexual	61 (89.7)	12 (19.7)	15 (24.6)	7 (11.5)	3 (4.9)	24 (39.3)	
LGBTQ	7 (10.3)	1 (14.3)	2 (28.6)	1 (14.3)	0 (0.0)	3 (42.9)	
Ever admitted (inpatient)							0.159
No	54 (83.1)	9 (16.7)	16 (29.6)	8 (14.8)	3 (5.6)	18 (33.3)	
Yes	11 (16.9)	4 (36.4)	1 (9.1)	0 (0.0)	0 (0.0)	6 (54.5)	
Defaulted from medical follow-up							0.632
No	37 (56.9)	8 (21.6)	9 (24.3)	6 (16.2)	2 (5.4)	12 (32.4)	
Yes	28 (43.1)	4 (14.3)	8 (28.6)	2 (7.1)	1 (3.6)	13 (46.4)	
Family history of eating disorder							0.785
No	56 (88.9)	11 (19.6)	14 (25.0)	8 (14.3)	3 (5.4)	20 (35.7)	
Yes	7 (11.1)	2 (28.6)	2 (28.6)	0 (0.0)	0 (0.0)	3 (42.9)	
Family history of psychiatric illness							0.012
No	46 (73.0)	10 (21.7)	14 (30.4)	8 (17.4)	3 (6.5)	11 (23.9)	
Yes	17 (27.0)	2 (11.8)	3 (17.6)	0 (0.0)	0 (0.0)	12 (70.6)	
Personal history of abuse							0.509
No	54 (81.8)	12 (22.2)	12 (22.2)	7 (13.0)	3 (5.6)	20 (37.0)	
Yes	12 (18.2)	1 (8.3)	5 (41.7)	1 (8.3)	0 (0.0)	5 (41.7)	
Reported trigger for ED symptomatology							0.028
Family stressor	21 (38.9)	1 (4.8)	3 (14.3)	4 (19.0)	1 (4.8)	12 (57.1)	
Social stressor	14 (25.9)	1 (7.1)	7 (50.0)	2 (14.3)	1 (7.1)	3 (21.4)	
Other stressor	19 (35.2)	7 (36.8)	6 (31.6)	2 (10.5)	0 (0.0)	4 (21.1)	

ED, eating disorders; AN, anorexia nervosa; BN, bulimia nervosa; BED, binge eating disorder; ARFID, avoidant restrictive/food intake disorder; OSFED, other specified eating disorder; DEBAs, disordered eating behaviors and attitudes; Triggers: Family-related stressor (parental discord, family pressure and loss); Social-related stressor (no voice, rejection, isolation, cyberbullying); Other (Sport injury).

The clinical features are described in Table 2 by ED category. At-risk behaviors observed found excessive exercise, calorie counting, and self-induced vomiting were the most prevalent. Excessive exercise was statistically associated with ED diagnosis (*p* = 0.029), being most prevalent among those with OSFED.

**Table 2:** The clinical features noted in patients by eating disorder category.

Feature	Total	AN	BN	BED	ARFID	OSFED	*p*-value
Common behaviors reported							
Self-induced vomiting	17 (100)	4 (23.5)	5 (29.4)	1 (5.9)	0 (0.0)	7 (41.2)	0.733
Excess exercise	24 (100)	8 (33.3)	5 (20.8)	0 (0.0)	0 (0.0)	11 (45.8)	0.029
Calorie counting	19 (100)	7 (36.8)	5 (26.3)	0 (0.0)	0 (0.0)	7 (36.8)	0.103
Laxative use	4 (100)	1 (25.0)	2 (50.0)	0 (0.0)	0 (0.0)	1 (25.0)	0.772
Diuretics use	1 (100)	0 (0.0)	0 (0.0)	0 (0.0)	0 (0.0)	1 (100.0)	0.780
CAM for weight loss	3 (100)	0 (0.0)	2 (66.7)	0 (0.0)	0 (0.0)	1 (33.3)	0.547
Presenting symptoms and signs							
Migraine	7 (100)	1 (14.3)	1 (14.3)	1 (14.3)	0 (0.0)	4 (57.1)	0.780
Syncope/near-syncope	15 (100)	4 (26.7)	0 (0.0)	0 (0.0)	0 (0.0)	11 (73.3)	0.003
Palpitations	11 (100)	4 (36.4)	0 (0.0)	1 (9.1)	1 (9.1)	5 (45.5)	0.172
Difficulty concentrating	27 (100)	8 (29.6)	4 (14.8)	2 (7.4)	2 (7.4)	11 (40.7)	0.192
Blurred vision	1 (100)	1 (100.0)	0 (0.0)	0 (0.0)	0 (0.0)	0 (0.0)	0.397
Abdominal pain	22 (100)	8 (36.4)	4 (18.2)	0 (0.0)	0 (0.0)	10 (45.5)	0.027
Cold intolerance	16 (100)	6 (37.5)	2 (12.5)	0 (0.0)	0 (0.0)	8 (50.0)	0.050
Alopecia	10 (100)	4 (40.0)	3 (30.0)	0 (0.0)	0 (0.0)	3 (30.0)	0.392
Constipation	10 (100)	4 (40.0)	2 (20.0)	0 (0.0)	0 (0.0)	4 (40.0)	0.327
Difficulty falling asleep	16 (100)	1 (6.3)	4 (25.0)	1 (6.3)	1 (6.3)	9 (56.3)	0.303
Nocturia/Difficulty staying asleep	5 (100)	0 (0.0)	0 (0.0)	0 (0.0)	0 (0.0)	5 (100.0)	0.061
Amenorrhoea	15 (100)	7 (46.7)	2 (13.3)	0 (0.0)	0 (0.0)	6 (40.0)	0.017

*p*-values represent Pearson chi-square analysis.

AN, anorexia nervosa; BN, bulimia nervosa; BED, binge eating disorder; ARFID, avoidant restrictive/food intake disorder; OSFED, other specified feeding or eating disorder; CAM, complementary alternative medicine.

The most prevalent symptoms observed were difficulty concentrating, abdominal pain or discomfort, difficulty falling asleep and cold intolerance. We found statistically significant associations with some symptoms such as migraine headaches, blurred vision and nocturia in relation to ED diagnoses, with greater prevalence in participants with anorexia nervosa.

Table 3 presents the frequency of psychological symptoms by ED category. The most prevalent symptoms were anxiety (*n* = 36; 52.9%), mood fluctuations (*n* = 30; 44.1%) and social withdrawal (*n* = 22; 32.4%). We found no statistically significant associations between ED category and psychological symptoms. Thirty two (47.1%) patients were placed on psychiatric medication, most commonly an SSRI (*n* = 24; 75%), followed by an atypical antipsychotic (*n* = 4; 12.5%).

**Table 3:** Frequency of psychological symptoms among participants by eating disorder category.

Symptom	Total *N* (%)	AN *n* (%)	BN *n* (%)	BED *n* (%)	ARFID *n* (%)	OSFED *n* (%)	*p*-value
Symptoms of anxiety	36 (100)	8 (22.2)	8 (22.2)	2 (5.6)	2 (5.6)	16 (44.4)	0.288
Substance misuse	7 (100)	0 (0.0)	4 (57.1)	1 (14.3)	0 (0.0)	2 (28.6)	0.260
Depressive symptoms	21 (100)	4 (19.0)	5 (23.8)	2 (9.5)	0 (0.0)	10 (47.6)	0.753
Social withdrawal	22 (100)	5 (22.7)	6 (27.3)	1 (4.5)	1 (4.5)	9 (40.9)	0.700
Irritability	13 (100)	2 (15.4)	2 (15.4)	0 (0.0)	1 (7.7)	8 (61.5)	0.140
Mood fluctuation	30 (100)	7 (23.3)	8 (26.7)	1 (3.3)	1 (3.3)	13 (43.3)	0.208
Suicidal ideation	17 (100)	2 (11.8)	5 (29.4)	2 (11.8)	0 (0.0)	8 (47.1)	0.540
Suicide attempt	5 (100)	0 (0.0)	2 (40.0)	1 (20.0)	0 (0.0)	2 (40.0)	0.759
Non-suicidal self-injury	9 (100)	1 (11.1)	2 (22.2)	1 (11.1)	0 (0.0)	5 (55.6)	0.713

AN, anorexia nervosa; BN, bulimia nervosa; BED, binge eating disorder; ARFID, avoidant restrictive/food intake disorder; OSFED, other specified feeding or eating disorder.

Table 4 presents the associations between therapy intervention characteristics and ED category. Readiness is an important concept in psychology, which refers to the motivation and willingness for change (Krampe et al., 2017). In this study, clinical judgement was used by the provider to determine readiness, which was informed by the transtheoretical model of change (Norcross and Lambert, 2018).

**Table 4:** Non-pharmacotherapy treatment and ED category.

Variable	Total	AN	BN	BED	ARFID	OSFED	*p-*value
Readiness for therapy							0.054
Very ready	2 (100)	1 (50.0)	0 (0.0)	1 (50.0)	0 (0.0)	0 (0.0)	
Ready	39 (100)	4 (10.3)	16 (41.0)	6 (15.4)	2 (5.1)	11 (28.2)	
Somewhat ready	15 (100)	5 (33.3)	1 (6.7)	1 (6.7)	0 (0.0)	8 (53.3)	
Therapy modality used							0.551
Individual	18 (100)	3 (16.7)	5 (27.8)	5 (27.8)	0 (0.0)	5 (27.8)	
Individual + family	35 (100)	7 (20.0)	11 (31.4)	2 (5.7)	2 (5.7)	13 (37.1)	
Individual but family planned	2 (100)	0 (0.0)	1 (50.0)	1 (50.0)	0 (0.0)	0 (0.0)	
Response to therapy							0.023
Client is pleased	38 (100)	7 (18.4)	12 (31.6)	6 (15.8)	1 (2.6)	12 (31.6)	
Client seems hopeful	5 (100)	0 (0.0)	1 (20.0)	2 (40.0)	0 (0.0)	2 (40.0)	
Pleased + occasional consult	7 (100)	2 (28.6)	2 (28.6)	0 (0.0)	2 (28.6)	1 (14.3)	
Unknown	10 (100)	4 (40.0)	2 (20.0)	0 (0.0)	0 (0.0)	4 (40.0)	
Continues to struggle	4 (100)	0 (0.0)	0 (0.0)	0 (0.0)	0 (0.0)	4 (100.0)	
Discontinued therapy	4 (100)	0 (0.0)	0 (0.0)	0 (0.0)	0 (0.0)	4 (100.0)	

ED, eating disorders; AN, anorexia nervosa; BN, bulimia nervosa; BED, binge eating disorder; ARFID, avoidant restrictive/food intake disorder; OSFED, other specified feeding or eating disorder.

We found that most participants stated they were ready to be involved in therapy related to their ED diagnosis (*p* = 0.054), with more participants with bulimia nervosa being ready or very ready for therapy. In addition, the response to therapy, which was based on feedback provided by the patient to the provider (feedback was based on a set of informal questions about what worked and what did not work), showed that most participants were pleased with the therapy they are presently receiving, significantly more so among patients with OSFED (*p* = 0.023).

Table 5 shows the multivariable logistic regression model examining the relationship between ED diagnoses and outcomes - hospital admission and default from follow-up. We found that compared to the other ED categories, participants diagnosed with anorexia nervosa had significantly higher odds of being admitted to hospital (OR = 13.53, *p* = 0.036) and less likely to default medical-care follow-up (OR = 0.14, *p* = 0.035). Those diagnosed with OSFED were at significantly higher odds of defaulting from medical follow-up (OR 26.01; *p* = 0.028).

**Table 5:** Predictors for hospital admission and defaulting from medical care.

Predictor	Hospital admission		Default from medical care	
	OR (95% CI)	*p*-value	OR (95% CI)	*p*-value
ED Diagnosis				
AN	13.53 (1.18–154.61)	0.036*	0.14 (0.02–0.87)	0.035*
BN	0.22 (0.02–3.13)	0.266	1.77 (0.32–9.84)	0.516
OSFED	1.01 (0.13–7.66)	0.993	2.62 (0.39–17.74)	0.323
ED type				
AN	Ref		Ref	
BN	1.77 × 10^−2^ (1.64 ×10^−7^, 1.86)	0.074	11.07 (0.65, 189.41)	0.097
BED	NA	NA	21.22 (0.53, 842.38)	0.104
ARFID	NA	NA	NA	NA
OSFED	0.32 (0.01, 8.37)	0.496	26.01 (1.43, 474.49)	0.028*
Sex				
Male	Ref		Ref	
Female	0.46 (0.02, 12.31)	0.644	NA	NA
Age category				
Early adolescence	Ref		Ref	
Late adolescence	0.18 (0.01, 3.14)	0.239	8.23 (0.71, 95.73)	0.092
Young adult	NA	NA	0.30 (0.02, 5.75)	0.425
Duration of having an ED	1.52 (0.73, 3.14)	0.262	1.31 (0.92, 1.85)	0.132
On psychotropic medication	1.56 (0.07, 33.16)	0.775	1.82 (0.25, 13.13)	0.551
Diagnosed with depression	0.12 (0.01, 1.85)	0.130	7.31 (0.96, 55.92)	0.055
Diagnosed with anxiety	1.75 (0.09, 33.61)	0.711	2.13 (0.31, 14.64)	0.441
History of abuse	60.36 (0.53, 6810.68)	0.089	0.97 (0.09, 10.68)	0.979
Family history of ED	0.64 (0.03, 13.39)	0.773	NA	NA
Family history of psychiatric illness	0.18 (0.00, 9.68)	0.398	0.08 (0.00, 1.14)	0.062

OR, odds ratio; CI, confidence interval; AN, anorexia nervosa; BN, bulimia nervosa; BED, binge eating disorder; ARFID, avoidant/restrictive food intake disorder; OSFED, other specified feeding or eating disorder; *p < 0.05. All models adjusted for sex, age at first ED physician visit, duration, depression, anxiety, history of abuse, family history of eating disorders, and family history of psychiatric disorders.

## Discussion

This review of the clinical features, management and note of adolescents and young adults living with an eating disorder in the Jamaican metropolis, Kingston, revealed two major findings, (1) an increased prevalence compared with a similar previous study (White and Gardner, 2002) with a persistent female preponderance and; (2) even though the majority of patients were managed as outpatients, as many as 1 in 6 AYAs (*n* = 11; 16.2%) were admitted for care, most commonly due to failed outpatient management. These findings challenge previous stereotypes by providing evidence that eating disorder cases are present not only in White industrialized countries but in all groups, in this study, among a predominantly Black ethnic population in a developing country.

We determined a tripling of patients (*n* = 68) diagnosed with an eating disorder two plus decades prior, over a similar time period to that reviewed by White and Gardener (*n* = 22) (White and Gardner, 2002). This increase is not unique to Jamaica, as similar findings are being reported globally (Da Luz et al., 2017; Kolar et al., 2016; Stark-Wroblewski et al., 2005; Morris et al., 2022). Notwithstanding, an increase in this developing context is of concern as the country is ill-equipped to care for individuals with EDs, with implications for a potential increase in the morbidity and mortality rates, potentiated by a lack of timely identification and intervention for those suffering from this illness.

We report a persistence of female predominance, but must highlight that males comprise 15% of the sample population, a novel and significant finding, almost doubling previous reports of 9% males (White and Gardner, 2002). This is in tandem with other local data which identified 17% of male adolescents as ‘at risk’ of developing an eating disorder’ using the EAT-26 screening tool (score >20), an increased prevalence of disordered eating behaviors over the decade prior (Harrison et al., 2020).

Furthermore, our findings may yet reflect underreporting, given the significant stigma that surrounds psychiatric diagnoses in Jamaica (Arthur et al., 2010; Arthur and Whitley, 2015), which may result in avoidance and rejection of an ED diagnosis, more so when it comes to males, as EDs are often referred to as a “female illness.” There is limited research presented on the characteristics of eating disorders among Black people, with most of the research focusing primarily on what is believed to be the most common type of eating disorder among Black people, Binge Eating Disorder (Yeboah et al., 2024), with AN rarely being reported in this population (Taylor et al., 2007). While not negating the occurrence of BED or associated symptoms among Black people (Dickens et al., 2024; Yoon et al., 2023), we caution against stereotypes being applied to ethnic groups regarding a subtype of ED, as this may result in those who fall outside of this category having challenges in being recognized (Sala et al., 2024) and treated appropriately (Moreno et al., 2023). Contrary to previous research, our study reports several classifications of ED being identified, with AN and OSFED (here comprised mainly of atypical AN) the most common subtypes identified in this ethnically diverse sample.

We report outcomes comparable to those in resource-competent spaces (Schmidt et al., 2025), however, we did have higher hospitalization rates (17%) than in some high-income countries (5.6%) (Lau et al., 2022). Patients suffering from an eating disorder may be admitted for a variety of medical or psychological indications - we identified failed outpatient management as the most common cause for admission in our sample. Noteworthy is that among the failed outpatient management, there were some instances of delayed intervention due to missed opportunities for diagnosis; this speaks to the need for ongoing training of healthcare providers in the identification of these patients.

There is a dearth of trained eating disorder professionals in Jamaica and the Caribbean. In 2009/2010, two such professionals (adolescent medicine physician and clinical psychologist) returned from overseas training and started a small ED management team, the first of its kind in Jamaica, co-opting other non-ED trained providers (psychiatrist, dietician) as needed. Our higher hospital admission rate may reflect a more cautious approach, given the small team available to manage both in-patients and out-patients. Delay in admission may result in further deterioration, and when later admitted in extremis, the in-patient management would likely be more challenging and more resource depleting.

Our findings add to the support of personalized treatment plans for patients and their families, which could be developed by a small, dedicated team in other similarly resource poor settings. However, with caution, as this model may not be sustainable, particularly as the prevalence of EDs increases, reaffirming the need to build capacity among healthcare providers in Jamaica and the Caribbean.

We face similar challenges to industrialized settings where eating disorder education and awareness remain relatively low (Moreno et al., 2023). Further, symptoms may be attributed to supernatural forces (Arthur et al., 2010; Arthur and Whitley, 2015) or may be mistakenly diagnosed as a physical illness or overlooked by physicians. This is cause for concern, especially considering that early detection and treatment of EDs improve prognosis (Nazar et al., 2017; Treasure et al., 2015). Sharing this evidence of the physical and psychological presentations and outcomes associated with eating disorders may serve to highlight the potential severity of the illness, as well as contradict the concept that the range of eating disorders do not exist among Black people, and more specifically among Jamaicans.

Previous studies have found identification of triggers to be particularly useful in aiding prevention and intervention. Two recent retrospective chart review studies in the USA (Lin et al., 2023) and Canada (Chen and Couturier, 2019) found that among a sample of White adolescents diagnosed with AN, factors such as environment, the internalization of the thin ideal, and health education triggered the onset of their illness. Our findings extended to include all ED categories, and highlighted the significant role of the family, with more than half of the patients identifying family as the trigger for their illness. The difference between our findings and that of industrialized White populations, underscores the relevance of culture-specific studies, especially among a vastly understudied sample. These findings are not surprising considering that Jamaica is classified a collectivist society (Hofstede, 2011) where the voices and opinions of the family are held in high regard and where there is evidence of authoritarian parenting styles and hierarchical power (UNICEF, 2010; Lipps et al., 2012).

In the Caribbean context, this parenting style is believed to have been influenced by a complex interplay of unique cultural, sociodemographic, and religious influences, which were born out of the history of slavery, colonialism, and independence. These influence the power dynamics between the adult and the child, where adults are revered with obedience and where there is pressure to conform (Payne, 1989). This “sociohistorical process” has been transferred from generation to generation, influencing families, and how members relate to others (Walkerdine et al., 2013). Within the Jamaican context, parents are described as disciplinarians and authority figures. In this authoritarian engagement, the parent emphasizes their hierarchical power and instils obedience in their children through coercive disciplinary measures such as corporal punishment, shaming, and humiliation, independent of socioeconomic category (Lewis, 2025; Green, 2021). This authoritarian parenting style has been associated with negative psychological, behavioral, emotional, and social outcomes among adolescents, including higher depression levels, low self-esteem, and low self-worth in teens (Jadon and Tripathi, 2017; Mandal et al., 2021; Jannah et al., 2022).

Concerningly, we also note a decrease in the mean age of participants compared to White and Gardener’s findings (White and Gardner, 2002), with our youngest patient in this study having been diagnosed at age ten years. Morris and colleagues report similar findings in Australian adolescents, with the incidence of EDs among children 5-13 years almost doubling over a decade, also with a reduction in the mean age at the time of diagnosis (Morris et al., 2022). The reduction in age of onset may be attributed to more frequent engagement and greater impact of social media and Westernization among Jamaican adolescents, as well as an increased capacity for early detection with the introduction of the aforementioned two subspecialty-trained providers in the healthcare system in the past decade.

The lack of significance of the onset of DEBA by ED category differs from previous studies (Slane et al., 2014; Robinson et al., 2020) that have found significance. An explanation could be the design of the study, as both were longitudinal studies compared to this cross-sectional study. While research on DEBAs and EDs in the English-speaking Caribbean region is in its infancy, we encourage researchers to also consider utilizing other methodologies, including longitudinal studies for a more fulsome understanding of its discourse. Our study found no significance between the mean age of onset for formal diagnosis. This may be attributed to the lack of knowledge and resources to implement routine assessment on body image, eating behaviors and mood, that while performed routinely in developed countries (Klein et al., 2021) does not appear to occur among healthcare providers in Jamaica.

Eating disorders are one of the most significant contributors to years of potential life lost (Chen and Couturier, 2019), and as the prevalence of EDs increases in Jamaica, we recommend that measures be instituted for prevention and treatment, including education of all stakeholders – adolescents/young adults, parents and healthcare providers. This will facilitate more careful screening for DEBAs during routine well visits, facilitating earlier diagnosis and intervention. Specialized multidisciplinary clinics, in- and out-patient treatment facilities, may be beneficial in creating an optimal treatment environment and perhaps decrease the risk of patients defaulting. Investment in the training of personnel specialized in the management of eating disorders, inclusive of dietitians, nurses, psychologists and physicians is expected to improve health outcomes for patients.

## Limitations of the study

This study was limited by missing data which is typical of retrospective chart reviews and may have introduced some measurement bias. Therefore, for the current study, we sought to do a complete case analysis based on non-missing data for our main outcome and main exposures. Some patients may also have defaulted from follow-up, limiting the determination of whether they resolved or deteriorated. Future research efforts could consider a prospective study design, which may offer an improved database for analysis, ensuring the collection of all relevant data, or the creation of an ED registry to better follow these patients. While we tried to identify all possible patients with an ED in the Kingston metropolitan region, there is the possibility that some patients may have been missed. This may be attributed to the lack of training and knowledge base as discussed earlier, but beyond healthcare professionals, further to the general society, often in denial of ED existence in Jamaica, assuming the myth of the stereotypes of the White and developed countries only, being the ones to suffer from this illness. Future work should be purposeful in educating the public as well as having standardized measures to ascertain the body image, mood and eating behaviors of young people in Jamaica. Despite these limitations, this study is of importance and relevance as it provides greater insight into the experience of patients with eating disorders and their management and outcome in Jamaica, even as these illnesses become increasingly prevalent in our population.

## Conclusion

This review of eating disorders among adolescents and young adults in Jamaica from 2010 to 2020 reveals an increased prevalence, tripling that from only 2 decades prior. As many as 1 in 6 patients required inpatient care in this resource-limited context, highlighting the urgent need for improved human resources, and the implementation of the necessary infrastructure for specialized care for these vulnerable patients to optimize outcomes. This study should raise awareness of eating disorders among healthcare providers in the region and provide evidence-based strategies to facilitate early diagnosis and management in a targeted and culturally appropriate manner.

## Data Availability

The datasets presented in this article are not readily available because it is a small dataset with a greater need to ensure anonymity. Requests to access the datasets should be directed to abigail.harrison@uwi.edu.
